# A Rare Case of Primary Midgut Volvulus Necessitating Extensive Bowel Resection in an Adult

**DOI:** 10.7759/cureus.4833

**Published:** 2019-06-05

**Authors:** Fatima Mustansir, Ayesha Farooq, Huma Baqir, Sejal A Gondal, Sadaf Khan

**Affiliations:** 1 Surgery, Aga Khan University, Karachi, PAK; 2 Psychiatry, Aga Khan University, Karachi, PAK

**Keywords:** mesenteric ischemia, midgut volvulus, short bowel syndrome, acute intestinal obstruction

## Abstract

A cause of acute intestinal obstruction in adults, midgut volvulus can be categorized into two types: primary type with no identifiable underlying cause, and secondary type that occurs in the presence of a predisposing condition such as, postoperative adhesions. Primary midgut volvulus can lead to bowel ischemia and necrosis, making an extensive bowel resection imminent. A potential consequence of bowel resection is short-bowel syndrome - a failure of digestion and absorption by the intestines, leading to malnutrition and other complications. As such, we report the diagnosis and management of primary midgut volvulus - a rare entity in adults - occurring in an adult patient.

## Introduction

Midgut volvulus is defined as the twisting of the bowel loops around the mesentery leading to vascular occlusion, ischemia, and eventually necrosis. Volvulus can be primary or secondary. In primary midgut volvulus, no underlying cause can be ascertained, whereas secondary midgut volvulus is due to congenital or acquired anatomic abnormalities [1]. We present a case of mesenteric ischemia secondary to midgut volvulus in a middle-aged lady, who then underwent an emergency laparotomy and extensive bowel resection (250 cm) at our hospital.

## Case presentation

A 50-year-old female presented to our hospital with generalized abdominal pain. The pain had been intermittent and was occurring spontaneously, every two to three weeks, for the last year. Her current episode spanned over 15 days and had gradually been increasing in intensity. The pain radiated to the back and was associated with three episodes of non-projectile, non-bilious vomiting. The pain had no association with meals. The review of systems revealed undocumented weight loss and reduced appetite. She had a history of Grade 3 ductal carcinoma of the left breast, treated with mastectomy and chemoradiotherapy 10 years previously. She had also undergone a laparoscopic cholecystectomy 15 years ago. Family history was not significant for any illness. At the time of presentation, she was being treated for Helicobacter pylori gastritis with triple therapy. There was no history of smoking, drug, or alcohol use.

At the time of presentation to the emergency room (ER), the patient was in severe pain. The patient was of short stature and lean, and was visibly distressed. At the time of presentation to the ER, she was hypotensive with a systolic blood pressure (SBP) ranging from 64 to 81 mm Hg and a diastolic blood pressure (DBP) of 30-40 mm Hg and tachycardic (Pulse = 131-150 bpm). She was afebrile. General physical examination revealed pallor and dehydration of conjunctival and oral mucosae. On abdominal examination, there was generalized tenderness but no guarding. Gut sounds were audible. The remaining physical examination was unremarkable. Resuscitation with intravenous fluids was initiated. A central venous catheter was placed to monitor fluid status and for the administration of norepinephrine. Tramadol was given to manage the pain and heparin sodium subcutaneously for deep venous thrombosis (DVT) prophylaxis.

Laboratory investigations revealed anemia (hemoglobin 8.9 gm/dl) and renal dysfunction (creatinine 2.2 mg/dl). Arterial blood gas suggested metabolic acidosis with respiratory compensation. The calculated serum osmolarity was 288 mOsm/kg and anion gap (corrected for albumin) was 26 (Table 1).

**Table 1:** Patient’s laboratory tests on admission. PT = Prothrombin Time; APTT = Activated Partial Thromboplastin Time; INR = International Normalized Ratio; Trop-I = Troponin-I; GGT = Gamma-Glutamyl Transferase; ALT = Alanine Aminotransferase; AST = Aspartate Aminotransferase; AP = Alkaline Phosphatase; BUN = Blood Urea Nitrogen.

Complete Blood Count		Basal Metabolic Profile		Others		Others		Arterial Blood Gases	
Hemoglobin (g/dl)	8.9	Sodium (mmol/L)	138	PT (seconds)	13.9	Direct bilirubin (mg/dl)	0.3	pH	7.24
Hematocrit (%)	26.8	Potassium (mmol/L)	4.3	APTT (seconds)	24.2	GGT (IU/L)	5	pCO_2_ (mmHg)	31
White blood cells (x10^9^/L)	5.9	Chloride (mmol/L)	103	INR	1.3	ALT (IU/L)	27	pO_2_ (mmHg)	208
Neutrophils (%)	77.1	Bicarbonate (mmol/L)	17	Trop-I	0.127	AST (IU/L)	43	Bicarbonate (mEq/L)	12.8
Lymphocytes (%)	12.7	BUN (mg/dl)	23	Albumin (g/dl)	2.4	AP (IU/L)	45	O_2_ saturation (%)	99.5
Platelets (x10^9^/L)	215	Cr (mg/dl)	2.2	Amylase (IU/L)	191	Calcium (mg/dl)	9	Base excess (mEq/L)	-13.2
		Fasting glucose (mg/dl)	83	Lipase (U/L)	25	Magnesium (mg/dl)	1.6		
				Total bilirubin (mg/dl)	0.5				

**Figure 1:**
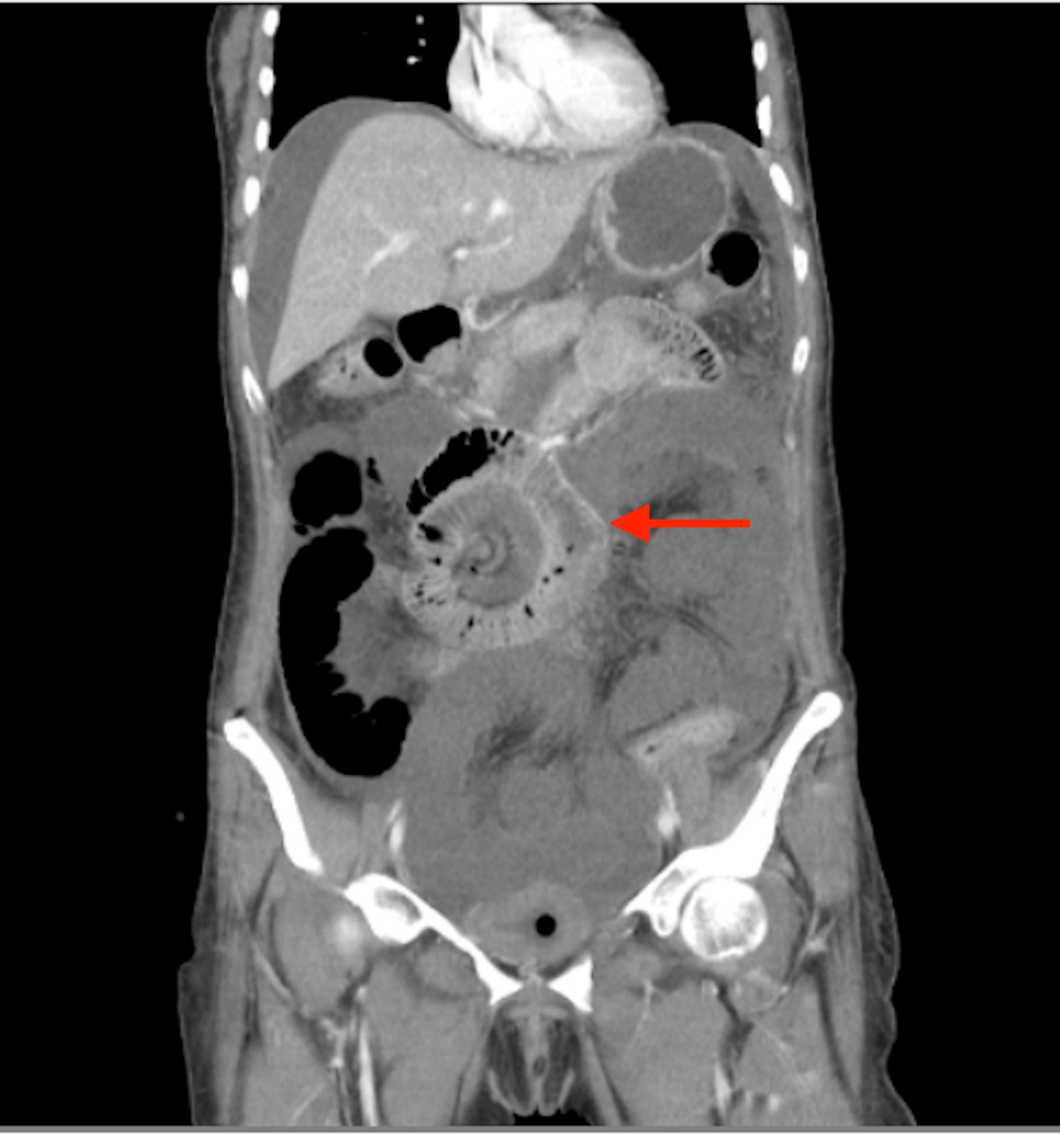
Computed tomography scan of the patient showing the classic “whirlpool” sign (red arrow).

Following volume resuscitation, she underwent a contrast-enhanced CT scan that showed significantly distorted bowel anatomy with abnormally dilated enhancing proximal and mid-jejunal loops. The presence of a midgut volvulus, affirmed by the presence of the whirlpool sign was noted (Figure 1). No congenital malrotation could be identified. The superior mesenteric artery was significantly attenuated representing occlusion, and there was non-enhancement of distal jejunal and ileal loops. These findings suggested mesenteric ischemia. There was no evidence of pneumatosis intestinalis or pneumoperitoneum. The liver was unremarkable.

**Figure 2:**
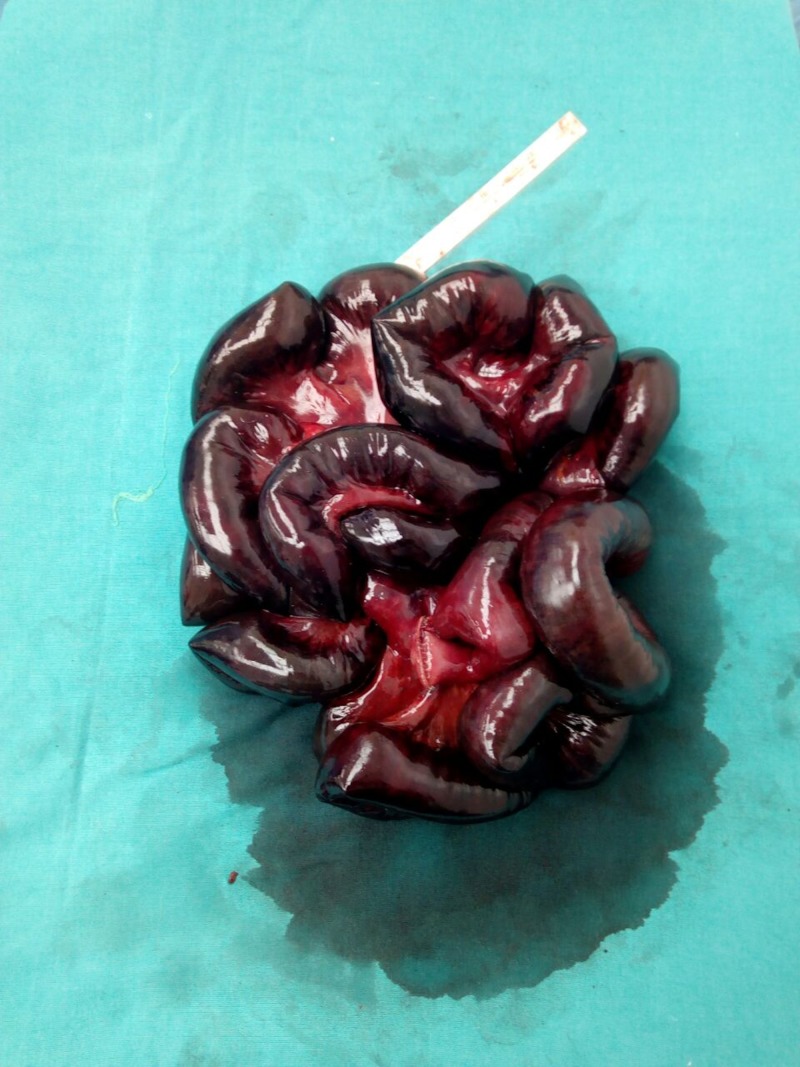
Approximately 250 cm of resected gangrenous small bowel.

Following successful resuscitation, an emergency laparotomy was performed. No underlying anatomic abnormality that could direct us towards the cause of the volvulus could be identified. Therefore, a presumptive diagnosis of primary midgut volvulus was made. Approximately 250 cm of gangrenous distal jejunum, ileum and cecum were resected (Figure 2). Seventy-five centimeters of duodenojejunum remained but had questionable viability in some areas. The abdomen was irrigated with three liters of fluid. The two stapled ends were left in place without an anastomosis. The fascia was not closed and the skin was approximated using towel clips.

The patient was shifted to the intensive care unit (ICU) and extubated. During the next 48 hours, she remained hemodynamically stable off vasopressors. She was afebrile, required minimal supplemental oxygen, and produced adequate urine. Anticoagulation was maintained via heparin sodium infusion.

She underwent a re-look laparotomy 48 hours later. Approximately 100 cc of thin murky fluid was found in the peritoneal cavity. The residual small bowel was viable with pulsatile mesenteric vessels throughout the remaining mesentery. There were no areas of necrosis identified. A side to side functional end to end stapled anastomosis was created between the mid jejunum and ascending colon. The fascia was closed and the skin left open.

She was again shifted to the ICU, and extubated a day later. Total parenteral nutrition (TPN) was initiated. The surgical wound was open and daily dressings were done. She was transferred to a special care unit (SCU) on day 5 of admission where she remained for a day before being shifted to the ward (day 6). She maintained adequate urine output and had multiple bowel movements every day from the sixth day of admission.

A complete nutritional assessment for this patient was done. She weighed 40 kg with a height of 155 cm, BMI of 18 kg/m^2^, and an ideal body weight of 48 kg. To meet her nutritional requirements, it was determined that she needed 1400-1800 kcal/day (35-45 kcal/kg/day). This was further split into 80 gm of protein, 175 gm of carbohydrates and 42 gm of fat. She was kept nil per oral (NPO) till the 2nd post-operative day, following the re-look procedure. During this time, she was kept on a TPN formula consisting of 10 gm of nitrogen, 200 gm of dextrose and 20 gm of fat, with a caloric value of 1130 kcal. Her TPN formula was subsequently adjusted to include 12 gm of nitrogen, 20 gm of fat, 260 gm of dextrose, 10 ml of multivitamin and 5 ml zinc sulfate, amounting to 1384 kcal. She was started on a soft diet on the 3rd post-operative day along with the new TPN formula. She was then progressed to a regular diet along with TPN. On the 6th post-operative day, TPN was discontinued and oral intake was supplemented with full-strength Ensure, at 200 ml twice a day. She was discharged on the 9th post-operative day on a regular diet, along with Ensure.

On her first visit to the outpatient clinic four days post-discharge, her weight was 35 kg and she was documented to have had multiple bowel movements (an average of four per day) at home. On subsequent monthly visits that continued for the next eight months, her weight remained fairly stable, going up to 36 kg; and she was found to be tolerating her diet well. She had no active complaints during these visits and her physical examination revealed adequate granulation tissue developing at the wound site.

## Discussion

Small bowel volvulus, although rarely encountered in the West (annual incidence is 1.7-5.7 per 100,000 population), is frequent in the East; the developing continents of Africa and Asia have an annual incidence of 24-60 per 100,000 population [2]. Small bowel volvulus can be differentiated into primary and secondary types. Of note, primary midgut volvulus occurs sporadically - no obvious anatomic cause can be identified upon laparotomy. As such, Vaez-Zadeh et al. have postulated that primary midgut volvulus can occur upon taking bulky meals after long periods of fasting, because this can induce bowel hypermotility and in the presence of a hypermobile mesentery can potentially lead to twisting [3]. One factor that can potentially lead to hypermobility is lack of fat surrounding the mesentery, the omentum, and the retroperitoneum [4]. As our patient was thin and lean, this could potentially have initiated volvulus in her.

Another theory (possible in our patient), to explain the findings of this case report, is that the volvulus in our patient was secondary to congenital adhesion bands or an internal hernia. Secondary midgut volvulus is initiated due to underlying congenital or acquired lesions that can lead to fixing of the bowel loop [5]. The most common cause of secondary midgut volvulus is postoperative adhesions. Examples of congenital lesions include intestinal malrotation and Meckel’s diverticulum [2]. Other predisposing conditions are a narrow mesenteric root, internal hernias, diverticulosis, congenital bands, jejunal lipomatosis, colostomy, fistula, pregnancy, endometriosis, abscess, mycobacterial disease, aneurysms, hematomas and tumors [2, 6]. In fact, midgut volvulus is the most common cause of bowel obstruction in pregnancy [6].

**Figure 3:**
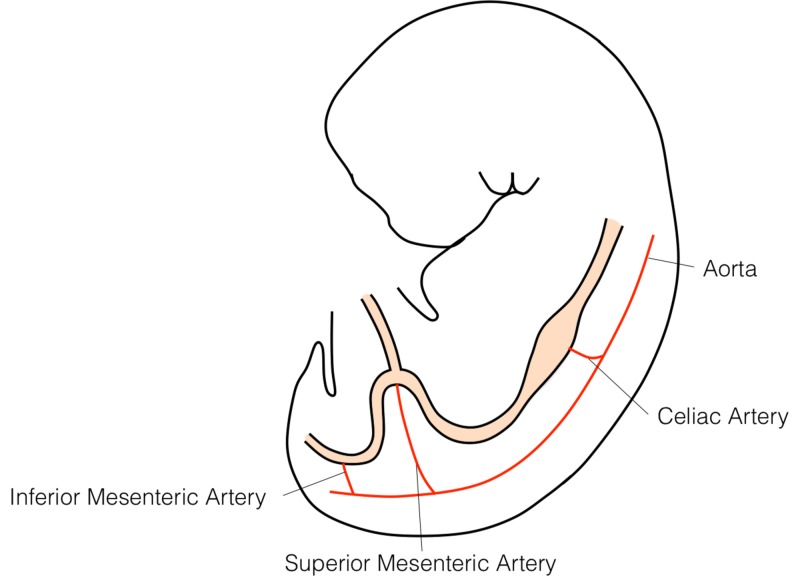
The three branches of the abdominal aorta, celiac trunk, superior mesenteric artery, and inferior mesenteric artery supply the foregut, midgut, and hindgut respectively.

The primitive gastrointestinal tract arises from the endoderm of the trilaminar embryo. In the 4th week of development, vascular pedicles arise from the gut tube that divides into foregut, midgut, and hindgut depending on blood supply (Figure 3). The superior mesenteric artery, a branch of the abdominal aorta, runs in the root of the mesentery of the small bowel and supplies the midgut (i.e., the length of the intestine from the distal duodenum to the proximal two-thirds of the transverse colon). The small bowel mesentery is a fan-shaped, fat-containing peritoneal fold that attaches the jejunum and ileum to the posterior abdominal wall. The mesentery runs obliquely in the abdomen from its proximal attachment on the left side of the L2 vertebral body to the right sacroiliac joint [5].

A volvulus occurs when the small bowel twists on the root of the mesentery leading to occlusion of the superior mesenteric artery. This leads to mesenteric ischemia and if the occlusion is sustained eventually necrosis of the intestines. The intestinal twisting can be seen on CT scan as the “whirlpool” sign that is pathognomonic for midgut volvulus. Figure 1 illustrates this phenomenon on a CT scan film of the patient.

The issue of greatest concern in our patient is intestinal failure secondary to loss of small bowel. Short bowel syndrome is defined as a type of intestinal failure that can occur after extensive bowel resection leading to a reduction in the surface area available for absorption. In adults, the causes of short bowel syndrome include cancer, mesenteric ischemia, inflammatory bowel disease, and trauma. The length of the adult small intestine varies from 360 to 600 cm. Of this, the duodenum measures 25-30 cm, whereas the remaining small intestine - extending from the ligament of Treitz to the ileocecal junction - measures 480 cm, with the proximal two-fifths being the jejunum and the remainder the ileum [7]. Resection of up to 50% of the intestinal length is generally well tolerated. If more than two-thirds of the intestine is resected, however, short bowel syndrome can occur.

In addition to length, the site of resection is also a predictor. For example, the jejunum is the primary site for nutrient absorption, but interestingly, is inefficient at absorbing water. Approximately 90% of the nutrient absorption (carbohydrates, proteins, fats, vitamins A, B, C, D, E, K, and folic acid) takes place in the proximal 100-150 cm of the jejunum [8]. Loss of the jejunum can thus lead to malnutrition and steatorrhea along with other manifestations of micronutrient deficiency. Unlike the jejunum, the ileum has many tight junctions and is efficient at absorbing water and concentrating the intestinal contents. The functions of the distal ileum involve vitamin B12 (via intrinsic factor) and bile salt absorption, and resection of the terminal ileum can lead to diarrhea, macrocytic anemia, and hyperoxaluric kidney stones (in short bowel syndrome, due to a reduction in the surface area available for fat absorption, intraluminal fats bind calcium and render it unavailable for oxalate, which can then not be excreted).

Interestingly, in the first two years after intestinal resection, the small intestine adapts to the loss of surface area by increasing the depth of crypts, villous hypertrophy, and proliferation of microvilli [9]. The muscular layers too increase in length and thickness. These absorptive changes are most prominent in the ileum while most changes that occur in the jejunum are increased brush-border enzyme and transporter activity [10].

The presence of the ileocecal valve predicts better intestinal function and weaning from parenteral nutrition. This was previously attributed to the fact that the valve independently prolongs transit time and prevents retrograde flux of colonic material, limiting bacterial overgrowth. New data, however, suggest that these protective functions are also related to the length of the terminal ileum and not just valve function. A very short, non-motile terminal ileum can overwhelm the ileocecal valve leading to bacterial overgrowth [11].

It has been shown that short-bowel syndrome can lead to many chronic complications including esophagitis [12], diarrhea [13], hepatic steatosis and cholestasis [14], cholelithiasis, electrolyte and micronutrient deficiencies [15], metabolic bone disease [16], nephrolithiasis [17], d-lactic acidosis [18], and catheter-related complications [19].

In our patient, of the small bowel, only 75 cm of duodenojejunum remained. The normal duodenum is of length 25-30 cm, meaning that only 40-55 cm of viable jejunum (normal length of jejunum = 160-200 cm) [20] remains in our patient. As jejunum is where most of the nutrient absorption occurs, we anticipate major macronutrient deficiencies in our patient due to carbohydrate, protein, and fat malabsorption. This can lead to steatorrhea. Vitamins A, B, C, D, E, and K too would be inadequately absorbed leading to deficiencies that can lead to manifestations such as night blindness (vitamin A is needed for the maintenance of specialized epithelia such as the Descemet’s membrane in the cornea), beri-beri (thiamine deficiency), pellagra (niacin deficiency), sideroblastic anemia (B6 deficiency), scurvy (defective collagen synthesis due to reduced vitamin C), osteomalacia (due to reduced vitamin D), delayed wound healing (vitamin K gamma-carboxylates factors II, VII, IX, and X). This can lead to a raised prothrombin time (PT). Ileal resection leads to a watery diarrhea and megaloblastic anemia due to B12 deficiency.

## Conclusions

Primary midgut volvulus is a cause of acute intestinal obstruction in adults. Although rare in Western countries, it is not uncommon in Eastern countries. A high degree of clinical suspicion along with imaging is needed to make a diagnosis. Delays in linkage to care can lead to bowel gangrene necessitating intestinal resection, following which patients need to be rehabilitated as they can develop short-bowel syndrome, potentially leading to chronic complications secondary to nutrient malabsorption.
